# Supplementary figures and images for: News source bias and sentiment on social media
Source: PLoS One. 2024 Oct 23;19(10):e0305148. doi: 10.1371/journal.pone.0305148 (PMC11498708; doi:10.1371/journal.pone.0305148)

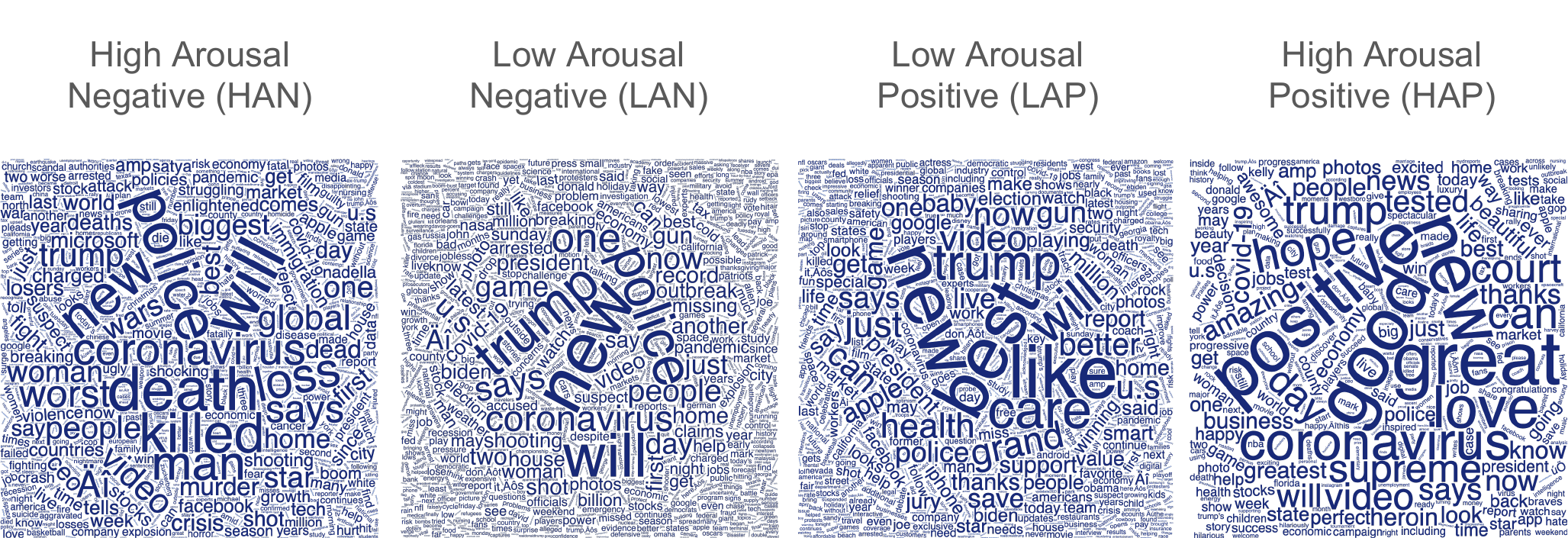

Supplement: S1 Fig — (TIF) [file pone.0305148.s001.tif]
