# Supplementary material for: News source bias and sentiment on social media
Source: PLoS One. 2024 Oct 23;19(10):e0305148. doi: 10.1371/journal.pone.0305148 (PMC11498708; doi:10.1371/journal.pone.0305148)
Supplement: S1 File — (DOCX) [file pone.0305148.s002.docx]

Supporting Materials for

News source bias and sentiment on social media

Brian Knutson, Tiffany W. Hsu, Michael Ko, Jeanne L. Tsai

Correspondence: [knutson@stanford.edu](mailto:xxxxx@xxxx.xxx)

**This file includes:**

- Supporting Methods
  - Supporting Method 1. Regression models for the influence of bias and year on affective content and virality
  - Supporting Method 2. Latent Dirichlet Analysis category key
- Supporting Results
  - Supporting Results 1. Regression analysis results for affective content prevalence
  - Supporting Results 2. Regression analysis results for affective content spread
  - Supporting Results 3.1-7. Affective content and virality by bias of posts in different news categories.

Supporting Methods

Supporting Method 1. Regression models for the influence of bias and year on affective content and spread

1. %Affect vs. |Bias|:

lm(formula = post_percentile ~ (Bias + Bias_abs), data = df.allinfo_byyear %>% filter(affect=="HAN")

2. %Affect x |Bias| x Year (coded as Year-2011) interaction:

lm(formula = post_percentile ~ (Bias + Bias_abs)*updated_year_num, data = df.allinfo_byyear %>% filter(affect=="HAN")

3. Affect (0=not contain, 1=contain) x |Bias| interaction:

lmer(formula = repost_percentile ~ HAN*Bias + HAN*Bias_abs + media + (1 | user_handle) + (1 | updated_year)

4. Affect (0=not contain, 1=contain) x |Bias| x Year (coded as Year-2011) interaction:
lmer(formula = repost_percentile ~ HAN*Bias*updated_year_num + HAN*Bias_abs*updated_year_num + media + (1 | user_handle))

Supporting Method 2. Latent Dirichlet Analysis news category key

1. Politics & Policy

- Key terms: Elections, Policymaking, Immigration, Climate change
- Description: Articles that are related to the government, political events, and governmental policies.

2. Sports

- Key terms: Soccer, Baseball, Basketball, Football, Tennis
- Description: Articles about competitive physical activities or games (including, soccer, baseball, etc.).

3. Health & Wellness

- Key terms: Ebola, Wellness, Medicine, Food, Drink, Exercise, Mental health, Sleep
- Description: Articles about health and well-being. These articles are related to aspects of a person’s physical or mental condition.

4. Business & Economy

- Key terms: Banking, Finance, Markets, Companies, Transportation, Media
- Description: Articles that are financial, business, and economy-related events. These articles are about the production and distribution of services (for example, articles about transportation, media, etc.).

5. Crime & Safety

- Key terms: Courts, Law, Investigations, Shootings, Prisons
- Description: Articles that are related to any aspect of the criminal justice system.

6. Culture & Lifestyle

- Key terms: Religion, Faith, Travel, Home & Garden, Weddings, Fashion, Beauty, Art, Design, Parenting, Entertainment, Books, Music, Movies, Gaming
- Description: Articles that are about any aspect of culture and lifestyle. These articles are about the beliefs, leisure and entertainment activities, and way of life for a person or group of people.

7. Science & Technology

- Key terms: Nature, Environment, Energy, Space
- Description: Articles that are about the study of the physical and natural world and its application for practical use (for example, technology).

**Supporting Results**

**Supporting Results 1. Regression analysis results for affective content prevalence.**

High Arousal Negative (HAN) content

|  | Estimate | Std. Error | df | t value | Pr(>\|t\|) |
| --- | --- | --- | --- | --- | --- |
| (Intercept) | 16.00298803 | 0.684347322 | 10.77865 | 23.384307 | 1.390440e-10 |
| Bias | -0.06835166 | 0.009992609 | 1683.02220 | -6.840221 | 1.103006e-11 |
| **\|Bias\|** | 0.37832391 | 0.016918704 | 1683.18221 | 22.361282 | 3.454036e-97 |

|  | Estimate | Std. Error | t value | Pr(>\|t\|) |
| --- | --- | --- | --- | --- |
| (Intercept) | 14.176317104 | 0.464175816 | 30.540835 | 0.000000e+00 |
| Bias | -0.036503577 | 0.019588035 | -1.863565 | 6.255624e-02 |
| \|Bias\| | 0.284761979 | 0.032532125 | 8.753255 | 4.925866e-18 |
| Year | 0.411455105 | 0.085882359 | 4.790915 | 1.805562e-06 |
| Bias*Year | -0.006790449 | 0.003554265 | -1.910507 | 5.623709e-02 |
| **\|Bias\|*Year** | 0.020085676 | 0.005960130 | 3.370006 | 7.685935e-04 |

Low Arousal Negative (LAN) content

|  | Estimate | Std. Error | df | t value | Pr(>\|t\|) |
| --- | --- | --- | --- | --- | --- |
| (Intercept) | 25.24137193 | 0.463242528 | 11.91265 | 54.488460 | 1.175678e-15 |
| Bias | 0.01913699 | 0.008240765 | 1683.09270 | 2.322235 | 2.033907e-02 |
| \|Bias\| | 0.04854189 | 0.013952434 | 1683.33884 | 3.479098 | 5.159177e-04 |

|  | Estimate | Std. Error | t value | Pr(>\|t\|) |
| --- | --- | --- | --- | --- |
| (Intercept) | 23.927233298 | 0.386671375 | 61.880022 | 0.000000e+00 |
| Bias | 0.044725592 | 0.016317379 | 2.740979 | 6.190189e-03 |
| \|Bias\| | 0.016508094 | 0.027100166 | 0.609151 | 5.425063e-01 |
| Year | 0.294447392 | 0.071542395 | 4.115705 | 4.046046e-05 |
| Bias*Year | -0.005444001 | 0.002960801 | -1.838692 | 6.613603e-02 |
| \|Bias\|*Year | 0.006608664 | 0.004964954 | 1.331062 | 1.833481e-01 |

Low Arousal Positive (LAP) content

|  | Estimate | Std. Error | df | t value | Pr(>\|t\|) |
| --- | --- | --- | --- | --- | --- |
| (Intercept) | 14.93314457 | 0.450180539 | 11.35618 | 33.171457 | 1.156063e-12 |
| Bias | -0.02383868 | 0.007325356 | 1683.08216 | -3.254269 | 1.159396e-03 |
| \|Bias\| | 0.11913105 | 0.012402639 | 1683.28385 | 9.605298 | 2.640953e-21 |

|  | Estimate | Std. Error | t value | Pr(>\|t\|) |
| --- | --- | --- | --- | --- |
| (Intercept) | 13.205052439 | 0.343896737 | 38.3983069 | 2.009797e-232 |
| Bias | -0.031419541 | 0.014512306 | -2.1650275 | 3.052523e-02 |
| \|Bias\| | 0.111541957 | 0.024102272 | 4.6278607 | 3.976482e-06 |
| Year | 0.384088247 | 0.063628181 | 6.0364486 | 1.932530e-09 |
| Bias*Year | 0.001521336 | 0.002633269 | 0.5777366 | 5.635190e-01 |
| \|Bias\|*Year | 0.001295287 | 0.004415717 | 0.2933356 | 7.693017e-01 |

High Arousal Positive (HAP) content

|  | Estimate | Std. Error | df | t value | Pr(>\|t\|) |
| --- | --- | --- | --- | --- | --- |
| (Intercept) | 7.42405836 | 0.315817701 | 17.10655 | 23.507417 | 1.830958e-14 |
| Bias | -0.04221522 | 0.008097115 | 1683.30063 | -5.213613 | 2.080716e-07 |
| \|Bias\| | 0.14479831 | 0.013708475 | 1683.88607 | 10.562686 | 2.690762e-25 |

|  | Estimate | Std. Error | t value | Pr(>\|t\|) |
| --- | --- | --- | --- | --- |
| (Intercept) | 6.804845370 | 0.379215732 | 17.9445228 | 4.843600e-66 |
| Bias | -0.082530293 | 0.016002754 | -5.1572557 | 2.801042e-07 |
| \|Bias\| | 0.131598383 | 0.026577631 | 4.9514715 | 8.101204e-07 |
| Year | 0.137828157 | 0.070162943 | 1.9644010 | 4.964735e-02 |
| Bias*Year | 0.008448840 | 0.002903712 | 2.9096682 | 3.665474e-03 |
| \|Bias\|*Year | 0.002445003 | 0.004869222 | 0.5021343 | 6.156385e-01 |

Neutral (Neu) content

|  | Estimate | Std. Error | df | t value | Pr(>\|t\|) |
| --- | --- | --- | --- | --- | --- |
| (Intercept) | 43.59372598 | 0.96468478 | 10.62645 | 45.189607 | 1.713025e-13 |
| Bias | 0.07789865 | 0.01344036 | 1683.04348 | 5.795876 | 8.097836e-09 |
| \|Bias\| | -0.46072105 | 0.02275622 | 1683.18768 | -20.245943 | 9.575540e-82 |

|  | Estimate | Std. Error | t value | Pr(>\|t\|) |
| --- | --- | --- | --- | --- |
| (Intercept) | 46.730583912 | 0.629760032 | 74.203794 | 0.000000e+00 |
| Bias | 0.061491735 | 0.026575624 | 2.313840 | 2.079603e-02 |
| \|Bias\| | -0.382269488 | 0.044137225 | -8.660932 | 1.073723e-17 |
| Year | -0.702157046 | 0.116518946 | -6.026119 | 2.057333e-09 |
| Bias*Year | 0.003556257 | 0.004822168 | 0.737481 | 4.609323e-01 |
| \|Bias\|*Year | -0.016391935 | 0.008086271 | -2.027131 | 4.280577e-02 |

**Supporting Results 2. Regression analysis results for affective content spread.**

High Arousal Negative (HAN) content

|  | Estimate | Std.Error | t value | df | Pr(>\|t\|) |
| --- | --- | --- | --- | --- | --- |
| (Intercept) | 49.2017246 | 0.16780566 | 293.20659 | 14.0057198 | 6.22E-28 |
| HAN | 1.45710618 | 0.02216976 | 65.7249426 | 29176683.7 | 0 |
| Bias | 0.00631489 | 0.00241112 | 2.61906859 | 196.868383 | 0.00950352 |
| \|Bias\| | -0.040831 | 0.00408557 | -9.9939468 | 190.611535 | 3.54E-19 |
| media | 2.31199883 | 0.01260102 | 183.477129 | 6034584.9 | 0 |
| HAN*Bias | -0.0090985 | 0.00106815 | -8.5179345 | 28632339.7 | 1.62E-17 |
| **HAN*\|Bias\|** | 0.17871406 | 0.00172258 | 103.747785 | 28562843.7 | 0 |

|  | Estimate | Std.Error | t value | df | Pr(>\|t\|) |
| --- | --- | --- | --- | --- | --- |
| (Intercept) | 49.9703481 | 0.05876668 | 850.317648 | 232.109248 | 0 |
| HAN | -1.2999776 | 0.05449218 | -23.856224 | 29816079.2 | 8.75E-126 |
| Bias | 0.0038258 | 0.00252617 | 1.51446669 | 313.541676 | 0.13091491 |
| Year | -0.1504645 | 0.00394029 | -38.186193 | 10008666.7 | 5.03384e-319 |
| \|Bias\| | -0.0641702 | 0.00420016 | -15.27805 | 281.716657 | 8.44E-39 |
| media | 2.03533395 | 0.01180438 | 172.421897 | 5441602.73 | 0 |
| HAN*Bias | 0.01321966 | 0.00255957 | 5.16479273 | 29491968.3 | 2.41E-07 |
| HAN*Year | 0.48725176 | 0.00875438 | 55.6580454 | 29893886.7 | 0 |
| Bias*Year | 4.19E-04 | 2.18E-04 | 1.92470544 | 2531340.85 | 0.05426633 |
| HAN*\|Bias\| | 0.32924413 | 0.00414504 | 79.4309233 | 29564554.5 | 0 |
| \|Bias\|*Year | 0.00418438 | 3.39E-04 | 12.3542802 | 2655360.86 | 4.63E-35 |
| HAN*Bias* Year | -0.004651 | 4.22E-04 | -11.030343 | 29640254.2 | 2.73E-28 |
| **HAN*\|Bias\|*Year** | -0.0269465 | 6.78E-04 | -39.762849 | 29715073.9 | 0 |

Low Arousal Negative (LAN) content

|  | Estimate | Std.Error | t value | df | Pr(>\|t\|) |
| --- | --- | --- | --- | --- | --- |
| (Intercept) | 49.1858671 | 0.1566741 | 313.937445 | 16.9141516 | 2.73E-33 |
| LAN | 0.69800109 | 0.01956257 | 35.6804331 | 29842864.7 | 8.06E-279 |
| Bias | 0.0074007 | 0.00236131 | 3.1341515 | 193.119055 | 0.00199234 |
| \|Bias\| | -0.0179691 | 0.00399987 | -4.4924156 | 186.762321 | 1.23E-05 |
| media | 2.27730971 | 0.01260718 | 180.635867 | 5473289.32 | 0 |
| LAN*Bias | -0.0275381 | 0.00102451 | -26.879237 | 29488028.3 | 3.86E-159 |
| LAN*\|Bias\| | 0.11347165 | 0.00161246 | 70.3719352 | 29630948.3 | 0 |

|  | Estimate | Std.Error | t value | df | Pr(>\|t\|) |
| --- | --- | --- | --- | --- | --- |
| (Intercept) | 49.6939381 | 0.05725305 | 867.970226 | 241.963184 | 0 |
| LAN | -0.0737965 | 0.04729984 | -1.5601853 | 29917375.4 | 0.1187161 |
| Bias | 0.01073667 | 0.00246888 | 4.34880377 | 330.353744 | 1.83E-05 |
| Year | -0.101285 | 0.00411411 | -24.618952 | 9829387.87 | 7.99E-134 |
| \|Bias\| | -0.0401937 | 0.00409918 | -9.8053025 | 295.337913 | 7.82E-20 |
| media | 2.00391982 | 0.01180987 | 169.681752 | 4714373.06 | 0 |
| LAN*Bias | -0.030759 | 0.00242262 | -12.696607 | 29785333.9 | 6.18E-37 |
| LAN*Year | 0.13938491 | 0.00756882 | 18.4156722 | 29927395.2 | 9.85E-76 |
| Bias*Year | -7.07E-04 | 2.23E-04 | -3.1753036 | 2244254.03 | 0.00149682 |
| LAN*\|Bias\| | 0.19194265 | 0.00382291 | 50.208474 | 29839885.1 | 0 |
| \|Bias\|*Year | 0.00399146 | 3.47E-04 | 11.4939602 | 2392445.92 | 1.42E-30 |
| LAN*Bias* Year | 3.22E-04 | 4.01E-04 | 0.80441959 | 29806319.7 | 0.4211547 |
| LAN*\|Bias\|* Year | -0.0143408 | 6.27E-04 | -22.868564 | 29849296.1 | 9.57E-116 |

Low Arousal Positive (LAP) content

|  | Estimate | Std.Error | t value | df | Pr(>\|t\|) |
| --- | --- | --- | --- | --- | --- |
| (Intercept) | 49.563017 | 0.15240583 | 325.204211 | 13.5676963 | 8.43E-28 |
| LAP | -1.3401577 | 0.02366675 | -56.626174 | 29745412.6 | 0 |
| Bias | -0.000764 | 0.00228921 | -0.3337209 | 191.781819 | 0.73895475 |
| \|Bias\| | 0.00762932 | 0.00387717 | 1.9677538 | 185.403835 | 0.05058764 |
| media | 2.25843853 | 0.01261007 | 179.097977 | 4988580.66 | 0 |
| LAP*Bias | 0.00842392 | 0.00121513 | 6.93252621 | 29288643.7 | 4.13E-12 |
| LAP*\|Bias\| | 0.03672277 | 0.00192097 | 19.1168173 | 29443907.9 | 1.83E-81 |

|  | Estimate | Std.Error | t value | df | Pr(>\|t\|) |
| --- | --- | --- | --- | --- | --- |
| (Intercept) | 49.6155762 | 0.05498191 | 902.398153 | 239.979224 | 0 |
| LAP | 0.02697547 | 0.05813595 | 0.46400658 | 29909530.4 | 0.64264304 |
| Bias | -0.006609 | 0.00237217 | -2.7860719 | 327.818047 | 0.00564568 |
| Year | -0.0172106 | 0.00388912 | -4.4253202 | 7628121.67 | 9.63E-06 |
| \|Bias\| | 0.0206071 | 0.00393974 | 5.2305739 | 293.511303 | 3.22E-07 |
| media | 1.97603375 | 0.01181132 | 167.300056 | 4261136.74 | 0 |
| LAP*Bias | 0.06681847 | 0.00288527 | 23.1584699 | 29778163.5 | 1.20E-118 |
| LAP*Year | -0.2308573 | 0.00918373 | -25.137639 | 29886635.3 | 1.94E-139 |
| Bias*Year | 8.53E-04 | 2.11E-04 | 4.04305819 | 1716739.51 | 5.28E-05 |
| LAP*\|Bias\| | -0.0670057 | 0.00459397 | -14.585577 | 29838202.6 | 3.47E-48 |
| \|Bias\|*Year | -0.0024331 | 3.30E-04 | -7.3773033 | 1783710.63 | 1.62E-13 |
| LAP*Bias* Year | -0.0101821 | 4.74E-04 | -21.474607 | 29711269.6 | 2.69E-102 |
| LAP*\|Bias\|* Year | 0.01786998 | 7.48E-04 | 23.8987386 | 29787343.9 | 3.17E-126 |

High Arousal Positive (HAP) content

|  | Estimate | Std.Error | t value | df | Pr(>\|t\|) |
| --- | --- | --- | --- | --- | --- |
| (Intercept) | 49.4739519 | 0.15249638 | 324.427059 | 13.8393499 | 2.93E-28 |
| HAP | -1.6002226 | 0.0320458 | -49.935492 | 29148915.3 | 0 |
| Bias | -0.0019068 | 0.00232032 | -0.8217921 | 192.805517 | 0.41221028 |
| \|Bias\| | 0.01824348 | 0.00393197 | 4.63978032 | 186.769109 | 6.55E-06 |
| media | 2.25940583 | 0.01260938 | 179.18446 | 5286073.21 | 0 |
| HAP*Bias | 0.01897098 | 0.00156114 | 12.1520084 | 27873281.5 | 5.60E-34 |
| HAP*\|Bias\| | -0.0305403 | 0.00252272 | -12.106094 | 28321971.6 | 9.81E-34 |

|  | Estimate | Std.Error | t value | df | Pr(>\|t\|) |
| --- | --- | --- | --- | --- | --- |
| (Intercept) | 49.7535117 | 0.05536308 | 898.67671 | 228.594498 | 0 |
| HAP | -1.6661894 | 0.07783047 | -21.40793 | 29742193.4 | 1.13E-101 |
| Bias | 0.00650562 | 0.0023781 | 2.73564234 | 307.033273 | 0.00658805 |
| Year | -0.0570622 | 0.00374651 | -15.230768 | 7139895.49 | 2.21E-52 |
| \|Bias\| | 0.01573464 | 0.00395601 | 3.97740157 | 276.642806 | 8.90E-05 |
| media | 1.97221741 | 0.01181072 | 166.985421 | 4429180.28 | 0 |
| HAP*Bias | -0.0343456 | 0.00366902 | -9.3609836 | 29314156.7 | 7.90E-21 |
| HAP*Year | 0.0142485 | 0.01223246 | 1.16481073 | 29836951.3 | 0.24409563 |
| Bias*Year | -0.0017555 | 2.04E-04 | -8.6152836 | 1598767.54 | 6.98E-18 |
| HAP*\|Bias\| | -0.0615533 | 0.00594746 | -10.349506 | 29513926.9 | 4.21E-25 |
| \|Bias\|*Year | 2.49E-04 | 3.19E-04 | 0.77943281 | 1654678.44 | 0.43572491 |
| HAP*Bias* Year | 0.00975817 | 5.99E-04 | 16.2964054 | 29472427.4 | 1.05E-59 |
| HAP*\|Bias\|* Year | 0.00584566 | 9.63E-04 | 6.07317736 | 29604412.8 | 1.25E-09 |

Neutral (Neu) content

|  | Estimate | Std.Error | t value | df | Pr(>\|t\|) |
| --- | --- | --- | --- | --- | --- |
| (Intercept) | 49.5078299 | 0.16098444 | 307.531778 | 15.1916033 | 3.05E-30 |
| NEU | -0.1279704 | 0.01759858 | -7.2716339 | 29117101.2 | 3.55E-13 |
| Bias | -0.002699 | 0.00245945 | -1.0974073 | 192.940811 | 0.27383107 |
| \|Bias\| | 0.07703172 | 0.00417078 | 18.4693703 | 187.405877 | 4.64E-44 |
| media | 2.31020054 | 0.01260555 | 183.268506 | 6217295.7 | 0 |
| NEU*Bias | 0.01724152 | 0.00095567 | 18.0413734 | 27948667.3 | 9.23E-73 |
| NEU*\|Bias\| | -0.2074015 | 0.00148407 | -139.75219 | 27956257.2 | 0 |

|  | Estimate | Std.Error | t value | df | Pr(>\|t\|) |
| --- | --- | --- | --- | --- | --- |
| (Intercept) | 49.1620689 | 0.061665 | 797.244232 | 261.059667 | 0 |
| NEU | 1.36741946 | 0.04176201 | 32.7431471 | 29798127.6 | 3.84E-235 |
| Bias | 0.01102805 | 0.00263014 | 4.1929523 | 342.016335 | 3.51E-05 |
| Year | 0.04964943 | 0.00455641 | 10.8966189 | 13123746.2 | 1.20E-27 |
| \|Bias\| | 0.10447362 | 0.00438923 | 23.8022593 | 311.847748 | 4.20E-72 |
| media | 2.04717702 | 0.01181065 | 173.333177 | 5842494.57 | 0 |
| NEU*Bias | -0.0132852 | 0.00219723 | -6.0463524 | 29309045.9 | 1.48E-09 |
| NEU*Year | -0.2741935 | 0.00681552 | -40.230788 | 29843813.3 | 0 |
| Bias*Year | -0.00277 | 2.35E-04 | -11.799749 | 3344807.39 | 3.92E-32 |
| NEU*\|Bias\| | -0.254801 | 0.00342901 | -74.307452 | 29415475.9 | 0 |
| \|Bias\|*Year | -0.0050692 | 3.73E-04 | -13.592698 | 3575944.85 | 4.44E-42 |
| NEU*Bias* Year | 0.00593203 | 3.71E-04 | 15.9764309 | 29311678.4 | 1.87E-57 |
| NEU*\|Bias\|* Year | 0.00869182 | 5.74E-04 | 15.147827 | 29446125.6 | 7.83E-52 |

**Supporting Results 3.1-7: Affective content prevalence and spread by news source bias for posts in classified news categories.**

*Supporting Result 3.1. Politics and policy:*

Affective content by news source bias

Affective virality by news source bias

*Supporting Result 3.2. Culture and lifestyle:*

Affective content by news source bias

Affective virality by news source bias

*Supporting Result 3.3. Health and wellness:*

Affective content by news source bias

Affective virality by news source bias

*Supporting Result 3.4. Sports:*

Affective content by news source bias

Affective virality by news source bias*

*Supporting Result 3.5. Business and economics:*

Affective content by news source bias

Affective virality by news source bias

*Supporting Result 3.6. Crime and safety:*

Affective content by news source bias

Affective virality by news source bias*

*Supporting Result 3.7. Science and technology:*

Affective content by news source bias

Affective virality by news source bias*
