# Supplementary material for: News source bias and sentiment on social media
Source: PLoS One. 2024 Oct 23;19(10):e0305148. doi: 10.1371/journal.pone.0305148 (PMC11498708; doi:10.1371/journal.pone.0305148)
Supplement: Attachment — Submitted filename: newsaff_responsetoreviewers_240901.docx [file pone.0305148.s003.docx]

PONE-D-24-21176

News source bias and sentiment on social media

PLOS ONE

Dear Dr. Knutson,

Thank you for submitting your manuscript to PLOS ONE. After careful consideration, we feel that it has merit but does not fully meet PLOS ONE’s publication criteria as it currently stands. Therefore, we invite you to submit a revised version of the manuscript that addresses the points raised during the review process.

While the two experts who reviewed your manuscript **considered it to be strong and have great potential for publication**, they also made suggestions regarding the *contextualization* and the illustration of your assessment and the verification of some *methodological* issues which I believe will help to improve your paper.

Please submit your revised manuscript by Sep 06 2024 11:59PM. If you will need more time than this to complete your revisions, please reply to this message or contact the journal office at [plosone@plos.org](mailto:plosone@plos.org). When you're ready to submit your revision, log on to https://www.editorialmanager.com/pone/ and select the 'Submissions Needing Revision' folder to locate your manuscript file.

Please include the following items when submitting your revised manuscript:

- A **rebuttal** letter that responds to each point raised by the academic editor and reviewer(s). You should upload this letter as a separate file labeled 'Response to Reviewers'.
- A **marked-up** copy of your manuscript that highlights changes made to the original version. You should upload this as a separate file labeled 'Revised Manuscript with Track Changes'.
- An **unmarked** version of your revised paper without tracked changes. You should upload this as a separate file labeled 'Manuscript'.

If you would like to make changes to your financial disclosure, please include your updated statement in your cover letter. Guidelines for resubmitting your figure files are available below the reviewer comments at the end of this letter.

If applicable, we recommend that you deposit your laboratory protocols in protocols.io to enhance the reproducibility of your results. Protocols.io assigns your protocol its own identifier (DOI) so that it can be cited independently in the future. For instructions see: <https://journals.plos.org/plosone/s/submission-guidelines#loc-laboratory-protocols>. Additionally, PLOS ONE offers an option for publishing peer-reviewed Lab Protocol articles, which describe protocols hosted on protocols.io. Read more information on sharing protocols at <https://plos.org/protocols?utm_medium=editorial-email&utm_source=authorletters&utm_campaign=protocols>.

We look forward to receiving your revised manuscript.

Kind regards,

Silvio Eduardo Alvarez Candido
Academic Editor
PLOS ONE

Journal requirements:

1. When submitting your revision, we need you to address these additional requirements.

Please ensure that your manuscript meets PLOS ONE's style requirements, including those for file naming. The PLOS ONE *style templates* can be found at 
https://journals.plos.org/plosone/s/file?id=wjVg/PLOSOne_formatting_sample_main_body.pdf and 
<https://journals.plos.org/plosone/s/file?id=ba62/PLOSOne_formatting_sample_title_authors_affiliations.pdf>.

We have now updated our style to meet the PLOS ONE style requirements throughout.

2. (1) In your Methods section, please include **additional information about your dataset and ensure that you have included a statement specifying whether the collection and analysis method complied with the terms and conditions for the source of the data**.

We now state in the Methods that: “Data collection and processing complied with the terms and conditions imposed by twitter.com (now x.com).” (line XXX)

(2) Please note that PLOS ONE has specific guidelines on code sharing for submissions in which author-generated code underpins the findings in the manuscript. In these cases, we expect **all author-generated code to be made available without restrictions upon publication of the work**. Please review our guidelines at https://journals.plos.org/plosone/s/materials-and-software-sharing#loc-sharing-code and ensure that your code is shared in a way that follows best practice and facilitates reproducibility and reuse.

We now provide our analysis code at: <https://osf.io/63pzy/>.

3. We note that the **grant information** you provided in the ‘Funding Information’ and ‘Financial Disclosure’ sections do not **match**.

When you resubmit, please ensure that you provide the correct grant numbers for the awards you received for your study in the ‘Funding Information’ section.

We have removed the updated funding information from the acknowledgments and now provide it in response to item 5 below.

4. Thank you for stating the following financial disclosure: 
 [This work was supported by NSF Grant 1732963, a Stanford Ethics, Society, and Technology Grant, and a Stanford HAI Faculty Seed Grant to JT and BK.].  
Please state what role the funders took in the study.  If the funders had no role, please state: ""*The funders had no role in study design, data collection and analysis, decision to publish, or preparation of the manuscript*."" 
If this statement is not correct you must amend it as needed. 
Please include this amended Role of Funder statement in your cover letter; we will change the online submission form on your behalf.

We now state in the Financial Disclosure that: “The authors have no conflicts of interest to declare. The funders had no role in study design, data collection and analysis, decision to publish, or preparation of the manuscript.” (line 587)

5. Thank you for stating the following in the Acknowledgments Section of your manuscript: 
[We thank Alexis Ceja for assistance with coding and classification, as well as Yiwei Luo, Jonas Schöne, the Tsai lab, and Spanlab for feedback on previous drafts. This work was supported by NSF Grant 1732963, a Stanford Ethics, Society, and Technology Grant, and a Stanford HAI Faculty Seed Grant to JT and BK.]
We note that you have provided funding information that is not currently declared in your Funding Statement. However, funding information should not appear in the Acknowledgments section or other areas of your manuscript. *We will only publish funding information present in the Funding Statement section of the online submission form*. 
Please remove any funding-related text from the manuscript and let us know how you would like to update your Funding Statement. Currently, your Funding Statement reads as follows: 
 [This work was supported by NSF Grant 1732963, a Stanford Ethics, Society, and Technology Grant, and a Stanford HAI Faculty Seed Grant to JT and BK.]

Please include your amended statements within your cover letter; we will change the online submission form on your behalf.

Thank you for this clarification -- we have removed this information from the Acknowledgments section and would like the Funding Statement to read, and now also mention this in our cover letter:

“This work was supported by NSF Grant 1732963, a Stanford Ethics, Society, and Technology Grant, and a Stanford HAI Faculty Seed Grant to JT and BK. The funders had no role in study design, data collection and analysis, decision to publish, or preparation of the manuscript.”

6. We note that you have indicated that there are restrictions to data sharing for this study. PLOS only allows data to be available upon request if there are **legal** or ethical **restrictions** on sharing data publicly. For more information on unacceptable data access restrictions, please see <http://journals.plos.org/plosone/s/data-availability#loc-unacceptable-data-access-restrictions>.

Before we proceed with your manuscript, please address the following prompts:

a) If there are ethical or legal restrictions on sharing a de-identified data set, please explain them in detail (e.g., data contain potentially identifying or sensitive patient information, data are owned by a third-party organization, etc.) and who has imposed them (e.g., a Research Ethics Committee or Institutional Review Board, etc.). Please also provide contact information for a data access committee, ethics committee, or other institutional body to which data requests may be sent.

We now provide summary data, but not raw data, which is prohibited by the current x.com data sharing policy. We also note this in the Data Availability statement (see below).

b) If there are no restrictions, please upload the **minimal anonymized data set necessary to replicate your study findings to a stable, public repository** and provide us with the relevant URLs, DOIs, or accession numbers. For a list of recommended repositories, please see
https://journals.plos.org/plosone/s/recommended-repositories. You also have the option of uploading the data as Supporting Information files, but we would recommend depositing data directly to a data repository if possible.

We now provide summary data in an OSF repository, as noted in the Data Availability statement:

“Summary data and analysis code supporting the reported findings are publicly available in an Open Science Framework repository (<https://osf.io/63pzy/>). Restrictions now apply to the raw data based on the data policy of x.com (formerly, twitter.com), which were used under license and so are no longer publicly available.” (line 591)

We will update your Data Availability statement on your behalf to reflect the information you provide.

7. Please **remove your figures from within your manuscript** file, leaving only the individual **TIFF/EPS image** files, **uploaded separately**. These will be automatically included in the reviewers’ PDF.

We have removed the figures from the manuscript file and uploaded them separately.

8. Please include **captions for your Supporting Information files at the end of your manuscript**, and update any in-text citations to match accordingly. Please see our Supporting Information guidelines for more information: http://journals.plos.org/plosone/s/supporting-information.

We now include captions for our Supporting Information files at the end of the manuscript and have updated in-text citations accordingly.

9. Please review your reference list to ensure that it is complete and correct. If you have cited papers that have been retracted, please include the rationale for doing so in the manuscript text, or remove these references and replace them with relevant current references. Any changes to the reference list should be mentioned in the rebuttal letter that accompanies your revised manuscript. If you need to cite a retracted article, indicate the article’s retracted status in the References list and also include a citation and full reference for the retraction notice.

We have reviewed and revised our references section to conform to PLOS ONE style.

[Note: HTML markup is below. Please do not edit.]

Reviewers' comments:

Reviewer's Responses to Questions

**Comments to the Author**

1. Is the manuscript technically sound, and do the data support the conclusions?

The manuscript must describe a technically sound piece of scientific research with data that supports the conclusions. Experiments must have been conducted rigorously, with appropriate controls, replication, and sample sizes. The conclusions must be drawn appropriately based on the data presented.

Reviewer #1: Yes

Reviewer #2: Yes

2. Has the statistical analysis been performed appropriately and rigorously?

Reviewer #1: Yes

Reviewer #2: Yes

3. Have the authors made all data underlying the findings in their manuscript fully available?

The [PLOS Data policy](http://www.plosone.org/static/policies.action#sharing) requires authors to make all data underlying the findings described in their manuscript fully available without restriction, with rare exception (please refer to the Data Availability Statement in the manuscript PDF file). The data should be provided as part of the manuscript or its supporting information, or **deposited to a public repository. For example, in addition to summary statistics, the data points behind means, medians and variance measures should be available**. If there are **restrictions** on publicly sharing data—e.g. participant privacy or **use of data from a third party**—those must be specified.

Reviewer #1: No

Reviewer #2: Yes

4. Is the manuscript presented in an intelligible fashion and written in standard English?

PLOS ONE does not copyedit accepted manuscripts, so the language in submitted articles must be clear, correct, and unambiguous. Any typographical or grammatical errors should be corrected at revision, so please note any specific errors here.

Reviewer #1: Yes

Reviewer #2: Yes

5. Review Comments to the Author

Please use the space provided to explain your answers to the questions above. You may also include additional comments for the author, including concerns about dual publication, research ethics, or publication ethics. (Please upload your review as an attachment if it exceeds 20,000 characters)

**Responses to Reviewers**

Reviewer #1: This work investigates whether the bias of the news source affects the creation of emotional content and its spread, and whether these differences have evolved over time. The authors analyzed the sentiment of 30 million tweets from 182 U.S. news sources, ranging from extreme left to extreme right bias, over a decade (2011-2020). The results indicate that biased news sources, whether left or right, produced more high-arousal negative emotional content compared to balanced sources. This high-arousal negative content also increased the likelihood of reposting for biased sources as opposed to balanced ones (but this trend did not hold for other types of emotional content). In recent years, the virality of high-arousal negative content rose, especially in balanced news sources and posts about politics.

I would like to commend the authors for a methodologically **robust** work. The study is **well thought out**. The results are perhaps not too surprising and mostly consistent with the pre-existing literature but it is still important to do the quantitative documentation of such trends. The **attention to detail and the robust methodology** is visible in many places of the paper like for instance when the authors triangulate their initial choice of news media bias ratings (from Ad Fontes) with another provider (AllSides). The LDA topic modeling to characterize the news sources sections driving the results was another nice touch. The statistical analysis is solid and some of the somehow counterintuitive results (like the increased high arousal negative affective content in balanced news sources) are an important contribution to the existing literature. I barely have much criticism to utter and my recommendation is to accept this paper with only minor revisions.

I would also like to commend again the authors because as a reader of their manuscript they come across as very **impartial** in their work, as it should be! This is unfortunately not always the case when reading these sorts of paper, and it feels like some authors have an agenda or some sort of viewpoint bias that they try to accommodate the results to. This is not the case at all in the current paper. It feels exquisitely neutral in that regard.

We deeply appreciate this encouraging feedback (particularly given that this is a new line of inquiry for us)!

Only a bit of minor constructive criticism for the authors at the risk of being pedantic. I think the weakest link in the paper are the visualizations where there is definitely room for improvement. All the detailed reporting about the different linear models used for the analysis in the text is fine but sometimes a compelling visualization can convey the point across very efficiently.

**Display more results graphically.**

We appreciate this opportunity to improve and augment the figures (and apologize for their originally suboptimal resolution). Specifically, we have now cleaned and re-rendered the existing figures, adding color to indicate affect type. Additionally, based on the feedback, we now have updated and integrated some of the figures from the Supplement into the manuscript (i.e., those depicting the validity of the bias ratings, a sentiment analysis flowchart, and a breakdown of the bias by time interaction).

The comments in the discussion section about the spread of negative affect news content through the population is highly relevant and important. Perhaps the authors should mention at least a sort of counter hypothesis: that people perhaps could be developing some sort of resistance or immunity after perhaps too much exposure to negative sentiment in their news diets and could be tuning out. I'm not saying that this is happening, but perhaps the authors should concede this as one of many potential hypotheses.

**Address potential alternative hypotheses.**

The notion that people habituate to high arousal negative content may have come from the figure showing that the difference in virality of high arousal negative content has narrowed over 10 years. This diminishing difference, however, is due to high arousal content becoming more viral for balanced sources rather than less viral for biased sources, increasing the overall virality of high arousal negative content over 10 years (which would be more consistent with sensitization than habituation). To clarify this increase, we now include a Figure from the Supplement (Figure 5) in the main manuscript which depicts this interaction. We also now clarify that both the overall production and virality of high arousal negative content increased over 10 years in the Discussion:

“The prevalence and spread of high arousal negative content in biased sources was not limited to current developments or events, since it generally increased over ten years. Further, the virality of high arousal negative content did not decrease over time for biased news sources, but rather increased over time for balanced news sources.” (line 487)

Finally, the usage of SentiStrength is well reasoned. I was just wondering whether an LLM like gpt-3.5-turbo would have a high degree of interrater agreement with the SentiStrength algorithm annotations, thus providing further evidence for the validity of the findings. But again, this is a bit pedantic.

**Consider the application of large language models to sentiment coding.**

We agree that these developments offer a promising set of methods for future inquiry (as further suggested by a new reference), and so now explicitly raise and recommend this research direction in the Discussion:

“These findings coincide with a rise in scientific research on affective phenomena due to advances in measurement (including sentiment analysis), which allow researchers to test qualitative predictions with quantitative tools [35]. Recently, researchers have developed even more precise and granular tools for sentiment analysis (e.g., Large Language Models), which might be leveraged to test the specificity as well as the generalizability of these findings [36].” (line 552)

To conclude, well done to the authors for a solid piece of academic work.

Thank you again for your thoughtful consideration and constructive suggestions.

Reviewer #2: The paper contributes to fill empirical gaps though solid assessments. It deals with a very **important issue** of how political bias influence the diffusion of news. The findings are not so surprising, but clearly **generate an empirical contribution**. I have only a couple of suggestions to improve the work after publication.

While the empirical analysis of the paper is excellent, the paper is a bit atheoretical to my taste. I miss recourse to more general ideas and mechanisms in the contextualization regarding existing explanations about why would biased theory spread more. In my view this would be important to better contextualize the paper in nearby discussions. This would be particularly important for the introduction of the paper.

**Provide theoretical context for the predictions in the Introduction.**

We now provide more theoretical context for the predictions in the Introduction:

“Neuroeconomic research further implies that highly aroused positive and negative affect which typically occur during anticipation of uncertain outcomes promote subsequent attentional engagement and even action [21].” (line 101)

We also return to address these hypotheses in the Discussion:

“This distinction between high and low arousal affect allowed us to test a prediction derived from an anticipatory affect model that highly arousing affective content would be more likely to capture attention and motivate behavior [21]. In the current findings, however, only high arousal negative affect showed a clear association with subsequent spread, extending previous findings associating negative affective content with news virality [15]. Notably, these patterns did not depend on the direction of news source bias, instead extending to biased news sources on both the left and the right [14]. These results add specificity to emerging findings which imply that negative affective content predominates and can drive virality in United States news media on both ends of the political spectrum [9,12,14,15].” (line 479)

Another point refers to the illustration of the findings. First, the definition of some figures may be improved. Also, in some parts the authors describe textually findings that could be nicely visually represented.

**Improve the definition of the figures, and visually represent more of the statistical findings.**

We now have improved the definition and detail of the existing figures and have added more figures (some from the supplement) to clarify the measures and statistical results. Specifically, we have now cleaned and re-rendered the existing figures, adding color to indicate affect type. Additionally, based on the feedback, we now have updated and integrated some of the figures from the Supplement into the manuscript (i.e., those depicting the validity of the bias ratings, a sentiment analysis flowchart, and a breakdown of the bias by time interaction).

I congratulate the authors for the excellent work.

We appreciate your careful consideration and constructive suggestions!

6. PLOS authors have the option to publish the peer review history of their article ([what does this mean?](https://journals.plos.org/plosone/s/editorial-and-peer-review-process#loc-peer-review-history)). If published, this will include your full peer review and any attached files.


If you choose “no”, your identity will remain anonymous but your review may still be made public.


**Do you want your identity to be public for this peer review?** For information about this choice, including consent withdrawal, please see our [Privacy Policy](https://www.plos.org/privacy-policy).

Reviewer #1: No

Reviewer #2: No
